# A Safer Alternative Bio-Repellent: Targeting Mosquito Odorant-Binding Proteins with Catnip-Derived Nepetalactones from *Nepeta cataria* Leaves

**DOI:** 10.3390/ijms27031572

**Published:** 2026-02-05

**Authors:** Tarawin Kiatlertpongsa, Siriporn Nonkhwao, Jarupa Charoenrit, Jirawat Saetan, Supawadee Duangprom, Sineenart Songkoomkrong, Prateep Amonruttanapun, Piyapon Janpan, Prasert Sobhon, Sakda Daduang, Napamanee Kornthong

**Affiliations:** 1 Research Unit in Innovative Marine Biotechnology and Natural Bio-Resources for Sustainable Health and Wellness, Thammasat University, Pathumthani 12120, Thailand; kiatlertpongsa@gmail.com (T.K.); siriphorn.nonkhaow@gmail.com (S.N.); jaa.charupa@gmail.com (J.C.); su.duangprom@gmail.com (S.D.); sineenartsong@gmail.com (S.S.); wanderer_sci@yahoo.com (P.A.); piyapon.ater@gmail.com (P.J.); 2 Ruamrudee International School, Minburi Campus, Bangkok 10510, Thailand; 3 Chulabhorn International College of Medicine, Thammasat University, Rangsit Campus, Pathumthani 12120, Thailand; 4 Division of Health and Applied Sciences, Faculty of Science, Prince of Songkla University, Hat Yai 90110, Songkhla, Thailand; jisaetan@gmail.com; 5 Department of Anatomy, Faculty of Science, Mahidol University, Bangkok 10400, Thailand; prasert.sob@mahidol.ac.th; 6 Division of Pharmacognosy and Toxicology, Faculty of Pharmaceutical Sciences, Khon Kaen University, Khon Kaen 40002, Thailand; sakdad@kku.ac.th

**Keywords:** *Nepeta cataria*, nepetalactones, odorant-binding proteins (OBPs), repellent, molecular docking, molecular dynamics simulation

## Abstract

The reliance on synthetic repellents such as N,N-diethyl-meta-toluamide (DEET) has raised health and environmental concerns, prompting the search for safer, plant-based alternatives. Catnip (*Nepeta cataria* L.), a rich source of iridoid monoterpenes, particularly nepetalactones, known for strong insect-repellent activity. However, their efficient extraction and molecular mechanisms in insect inhibition remains challenging. This study examined the chemical composition, protein–ligand interactions, and safety profiles of nepetalactones in comparison with DEET, with particular focus on mosquito odorant-binding proteins (OBPs) from *Anopheles gambiae* (*Agam*OBP), *Culex quinquefasciatus* (*Cqui*OBP), and *Aedes aegypti* (*Aaeg*OBP). GC–MS/MS analysis identified nepetalactone isomers as the predominant constituents in catnip extracts obtained via steam distillation and olive oil extraction from dried leaves. Molecular docking results indicated that *cis,cis*-, *cis,trans*-, and nepetalactone isomers exhibited higher binding affinities toward the target OBPs than DEET. Furthermore, molecular dynamics simulations confirmed that all nepetalactone–OBP complexes exhibited stable conformations characterized by low average RMSD values and persistent hydrogen bond formation. Notably, *cis,trans*-NL–*Aaeg*OBP, NL–*Aaeg*OBP, and *cis,cis*-NL–*Agam*OBP complexes displayed lower binding free energies (Δ*G_MM-PBSA_*) compared to DEET. These findings suggest that nepetalactones stabilize OBP–ligand interactions while inducing subtle conformational flexibility, potentially disrupting mosquito odorant recognition in a manner distinct from DEET. ADMET predictions indicated that nepetalactones exhibit favorable absorption, distribution, and safety profiles with reduced predicted toxicity compared to DEET. Collectively, these results establish nepetalactones as promising candidates for the development of effective, safe, and sustainable plant-based repellents.

## 1. Introduction

Vector-borne diseases remain a significant global health challenge, with mosquitoes such as *Anopheles gambiae*, *Culex quinquefasciatus*, and *Aedes aegypti* serving as primary vectors for malaria, filariasis, dengue, Zika, and chikungunya [1,2]. The frontline strategy for reducing disease transmission has been the use of synthetic repellents, among which N,N-diethyl-meta-toluamide (DEET) remains the gold standard, offering broad-spectrum efficacy against multiple insect species [3]. Nevertheless, despite its effectiveness, DEET has raised safety concerns, along with ongoing debates about its mechanisms of action, including neurological interactions and potential molecular targets beyond simple olfactory masking [4,5,6,7]. Additionally, its precise mode of action remains under investigation; multiple studies support the idea that DEET influences mosquito behavior by modulating olfactory and chemosensory pathways. Furthermore, the growing interest in eco-friendly and biodegradable alternatives underscores the urgent need for plant-derived repellents that combine efficacy with enhanced safety profiles [8,9]. One promising candidate is catnip (*Nepeta cataria* L.), a perennial herb of the Lamiaceae family. Catnip is well known for its natural mosquito-repellent properties and is also associated with a chemical defense mechanism in felids [10]. Moreover, traditionally valued for its medicinal properties and its euphoric effects in felines, catnip has recently attracted scientific attention for its strong insect-repellent activity [11]. Its essential oil contains iridoid monoterpenes, particularly nepetalactones, stereoisomers such as *cis*,*trans*-nepetalactone, *cis*,*cis*-nepetalactone, and nepetalactone that account for up to 80% of the volatile fraction [12]. Additionally, catnip oil significantly induced escape behavior in such as *A. aegypti* in an automated excito-repellency assay, demonstrating both irritant and repellent effects in a concentration-dependent manner [13]. However, extracting nepetalactone and its derivatives is a challenging and time-consuming process. Notably, these compounds have demonstrated repellent activities against a range of arthropods, including mosquitoes, flies, ticks, and cockroaches, with some studies reporting efficacy equal to or exceeding that of DEET [13,14]. Nepetalactones activate the transient receptor potential ankyrin 1 (TRPA1) receptor in *Aedes aegypti*, an ion channel involved in chemical nociception, thereby inducing avoidance behavior, whereas catnip does not activate human TRPA1 [11].

Nevertheless, there were studies that revealed that mosquito odorant-binding proteins (OBPs) play a central role in insect olfaction by binding and transporting semiochemicals to olfactory receptors, thereby regulating host-seeking behavior [15,16,17]. Structural and biochemical evidence further indicates that OBPs possess flexible binding pockets capable of accommodating diverse ligands, and disruption of OBP–odorant interactions represents a key mechanism through which repellents interfere with host detection [18,19]. Previous studies have identified Odorant Binding Protein 1 from *Anopheles gambiae* (*Agam*OBP1) as a molecular target of DEET and have since investigated its interactions with other repellents across multiple mosquito species and insects, indicating that this protein may serve as a common target for insect repellents [20,21,22]. Comparative analyses of DEET and nepetalactones with mosquito OBPs can provide critical mechanistic insights into repellent action and inform the rational design of next-generation bio-repellents. Although nepetalactones from *N. cataria* have been widely reported as effective mosquito repellents, most previous studies have primarily focused on behavioral assays or isolated docking analyses [23,24]. To date, a comprehensive, comparative, and dynamic evaluation of nepetalactone interactions across multiple mosquito OBPs, integrating extraction efficiency, computational binding prediction, and safety assessment, remains limited.

In this study, we first evaluated multiple extraction techniques to identify the most efficient method in terms of yield, procedural simplicity, and the use of environmentally friendly solvents. We then investigated the comparative binding affinity and conformational effects of nepetalactone isomers and DEET on mosquito OBPs, providing molecular-level insight into how nepetalactones may induce flexibility or stabilization within OBP binding pockets and thereby contribute to repellent efficacy. In parallel, we predicted toxicological properties, supporting the potential of nepetalactones as safer alternatives to conventional synthetic repellents [24].

To address these gaps, we employed a multifaceted framework integrating gas chromatography–tandem mass spectrometry (GC–MS/MS) for phytochemical profiling, molecular docking, and replicated molecular dynamics simulations to evaluate interactions with OBPs from *A. gambiae* (*Agam*OBP, PDB ID: 3N7H), *C. quinquefasciatus* (*Cqui*OBP, PDB ID: 3OGN), and *A. aegypti* (*Aaeg*OBP, PDB ID: 3K1E), and ADMET prediction to assess pharmacokinetic and safety profiles. By combining chemical, computational, and toxicological perspectives, this study provides a comprehensive and mechanistic evaluation of catnip-derived nepetalactones as potential bio-repellents. Thus, these findings not only expand our mechanistic understanding of insect repellency but also contribute to the development of safer, sustainable, and plant-based alternatives to synthetic repellents.

## 2. Results

### 2.1. GC–MS/MS Identification of Catnip Extracts

The compound composition of *Nepeta cataria* extracts obtained via three different extraction techniques, including stream distillation (SDE) and maceration methods, including fresh leaf in olive oil extraction (FOE) and dried leaf in olive oil extraction (DOE), was analyzed using GC–MS/MS (Figure S1) [25]. The findings demonstrated that the extraction approach exerted a pronounced influence on qualitative profiles of volatile and semi-volatile constituents, including hydrocarbons, oxygenated volatiles, esters, phenolics, terpenoids, and iridoid lactones (Table 1).

**Figure 1:**
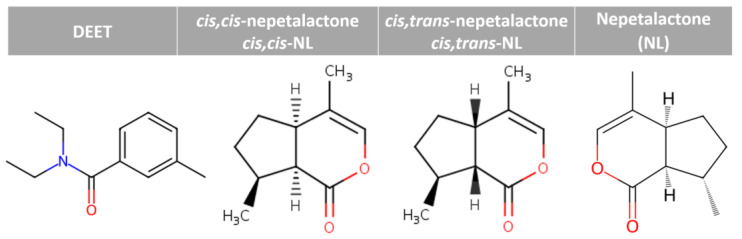
Chemical structures of DEET and nepetalactones.

Across both leaf macerations in oil extraction conditions, the predominant compound was identified as butylated hydroxytoluene (BHT), with relative abundances of 31.80% in FOE and 18.22% in DOE. In DOE, caryophyllene (0.12%) was identified as the only terpene in this fraction. Next, hydrocarbon constituents, particularly alkanes such as undecane, 2,6-dimethylnonane, and heptadecane, were also consistently abundant. FOE exhibited a higher alkane content (26.97%) compared to DOE (22.80%). Notably, the olive oil-based extracts from both FOE and DOE contained approximately 41.53% alkanes, contributing to their overall composition. Oxygenated aliphatic alcohols were detected in appreciable relative area, with 3,7-dimethyl-1-octanol reaching 4.11% and 2-isopropyl-5-methyl-1-heptanol reaching 3.48%, both enriched in FOE. Esters such as tetrahydrogeranyl formate were also relatively abundant FOE (4.48%). However, nepetalactones were completely absent, likely due to the high-water content in fresh leaf conditions. FOE and DOE favored the recovery of oxygenated volatiles, esters, and phenolic antioxidants. Most notably, DOE and SDE yielded iridoid lactones, recognized as the hallmark bioactive constituents of catnip with established insect-repellent properties (Figure 1). GC–MS/MS can tentatively identify *cis,cis*- and *cis,trans*-derivatives based on their differing retention times, as compared with reference standards and literature reports [26]. SDE fraction included *cis,cis*-nepetalactone; *cis,cis*-NL (52.80%) and *cis,trans*-nepetalactone; *cis,trans*-NL (47.20%), and as major compositions. DOE fraction found *cis,cis*-NL (4.90%), *cis,trans*-NL (1.80%), and nepetalactone; NL (0.24%).

Interestingly, DOE produced the broadest spectrum of compounds, particularly nepetalactone isomers, highlighting it as a green and efficient method for isolating bio-repellent constituents from *N. cataria*. Notably, olive oil maceration also represents a simple, eco-friendly, and rapid extraction method, with potential synergistic contributions from hydrocarbons, oxygenated volatiles, and phenolic compounds [27]. Nevertheless, the highest yields of nepetalactones were obtained from steam distillation.

### 2.2. Structural Analysis of OBP Receptors

**Figure 2:**
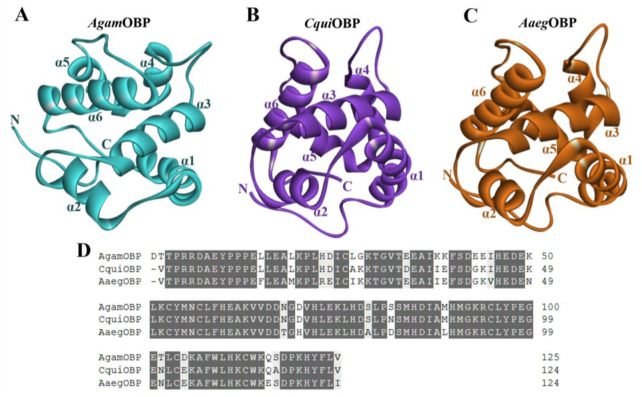
(**A**–**C**) Three-dimensional structures of *Anopheles gambiae* OBP (*Agam*OBP, PDB ID: 3N7H), *Culex quinquefasciatus* OBP (*Cqui*OBP, PDB ID: 3OGN), and *Aedes aegypti* OBP (*Aaeg*OBP, PDB ID: 3K1E), respectively. (**D**) Amino acid sequence alignment of the OBPs. Dark gray and light gray highlights indicate amino acid identity and similarity, respectively.

The three-dimensional structures and mature amino acid sequences of *Anopheles gambiae* OBP (*Agam*OBP, PDB ID: 3N7H), *Culex quinquefasciatus* OBP (*Cqui*OBP, PDB ID: 3OGN), and *Aedes aegypti* OBP (*Aaeg*OBP, PDB ID: 3K1E) were obtained from the RCSB Protein Data Bank (RCSB PDB) to characterize their structural organization and suitability as receptor targets for bio-repellent docking [28]. All three proteins adopted the canonical OBP fold, consisting of six α-helices (α1–α6) that surround a central hydrophobic binding pocket (Figure 2A–C). This conserved helical scaffold is stabilized by three disulfide bridges, which are essential for maintaining the structural integrity of the ligand-binding cavity. Despite their overall structural similarity, subtle differences were observed in the orientation of helices α4–α6 and the dimensions of the binding pocket, which may contribute to variation in ligand recognition across mosquito species. *Agam*OBP displayed a relatively compact binding cavity, while *Cqui*OBP presented a slightly wider pocket, and *Aaeg*OBP exhibited an elongated hydrophobic groove, suggesting differential adaptability to diverse ligands. Sequence alignment of the three OBPs revealed a high degree of conservation. *Agam*OBP showed 90.32% and 82.26% sequence identity with *Cqui*OBP and *Aaeg*OBP, respectively, while *Cqui*OBP shared 87.10% identity with *Aaeg*OBP. All three proteins also conserved six cysteine residues forming disulfide bridges, along with multiple hydrophobic residues that line the ligand-binding channel. These conserved features underscore their functional role in stabilizing protein folding and ligand interaction (Figure 2D).

### 2.3. Molecular Docking of DEET and Nepetalactone Isomers with OBPs

**Figure 3:**
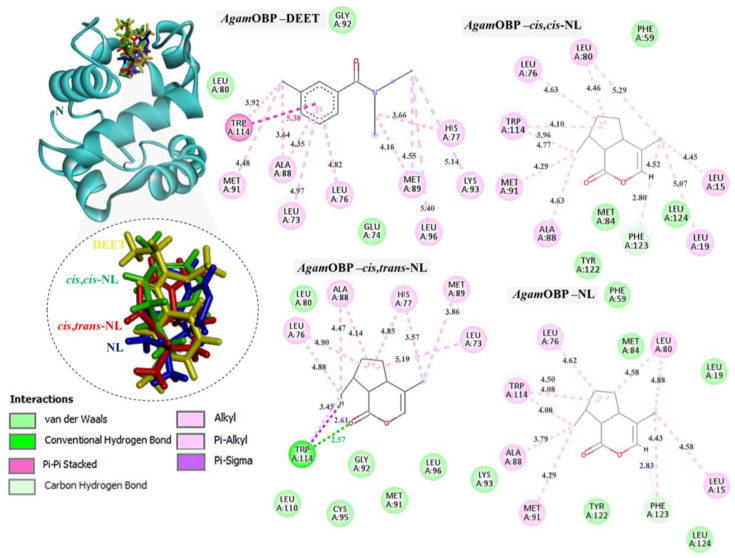
Complexes of *Agam*OBP with DEET, *cis,cis*-NL, *cis,trans*-NL, and NL are represented in yellow, green, red, and blue sticks, respectively. The dotted circles highlight the magnified superimposition of DEET and nepetalactone ligands after docking. The right panel shows the 2D schematic interaction diagrams of each complex.

Docking of DEET and nepetalactone derivatives against *Agam*OBP, *Cqui*OBP, and *Aaeg*OBP was performed using the CB-Dock2 server, and the complex with the lowest Vina binding energy was selected as the most efficient (Table 2) [29,30]. DEET, used as a reference synthetic repellent, exhibited moderate and relatively uniform binding scores of −6.4, −6.3, and −6.2 kJ/mol with *Agam*OBP, *Cqui*OBP, and *Aaeg*OBP, respectively. In contrast, all nepetalactone isomers showed stronger predicted affinities than DEET across all three OBPs. Notably, nepetalactones displayed particularly strong interactions with *Agam*OBP, with docking scores of −6.6 kJ/mol for each isomer. These complexes formed stable hydrogen bonds and exhibited tighter interactions with the key residue Trp114 compared to DEET (Figure 3). For *Cqui*OBP, *cis,cis*-NL, *cis,trans*-NL, and NL showed docking scores of −6.6, −6.7, and −6.5 kJ/mol, respectively, outperforming DEET (−6.3 kJ/mol). Similarly, nepetalactones demonstrated stronger binding to *Aaeg*OBP, with scores of −7.0, −6.7, and −7.0 kJ/mol. In these complexes, *cis,cis*-NL and *cis,trans*-NL formed hydrogen bonds, while NL showed tighter binding through π–σ interactions, suggesting a stable and consistent binding profile across receptor types.

Analysis of binding pocket volumes revealed structural variability among the OBPs. *Agam*OBP possessed the smallest cavity (1159 Å^3^), *Cqui*OBP the largest (1643 Å^3^), and *Aaeg*OBP an intermediate size (1284 Å^3^). These architectural differences may influence ligand accommodation and selectivity, with larger or more flexible pockets potentially enabling stronger stabilization of bulky compounds such as nepetalactones.

Interestingly, molecular docking was performed with ligands identified across all fractions, including FOE, DOE, and olive oil, against the *Agam*OBP, *Cqui*OBP, and *Aaeg*OBP receptors. Several ligands, such as 2,6,10-trimethyldodecane, 2-methyldecahydronaphthalene, butylated hydroxytoluene, and *trans*-4a-methyl-decahydronaphthalene, exhibited equal or higher binding affinity to all receptors compared with DEET and nepetalactones (Table S1). This finding indicates that *N. cataria* leaf extract in olive oil enhances or synergizes the repellent effect [31].

The binding interactions of DEET and the three nepetalactones with the three OBP receptors were characterized by a combination of hydrophobic contacts, hydrogen bonds, van der Waals forces, π–π stacking, π–σ, and alkyl interactions. For *Agam*OBP, DEET established multiple hydrophobic contacts (alkyl and π–alkyl) with residues Leu73, Ala88, and Met89, as well as van der Waals interactions with Leu80, Glu74, and Gly92. The aromatic ring of DEET formed a key π–π stacking interaction with Trp114, suggesting a central role of this residue in ligand stabilization. *Cis,cis*-NL exhibited strong van der Waals interactions with Phe59, Met84, Tyr122, and Leu124, alongside hydrophobic contacts involving Leu76, Leu80, Ala88, Met91, and Trp114. A hydrogen bond was also observed with Phe123. Similarly, nepetalactone established a hydrogen bond with Phe123 and extensive hydrophobic interactions with Leu76, Leu80, Ala88, Met91, and Trp114, complemented by van der Waals interactions with Leu19, Met84, Tyr122, and Leu124. *Cis,trans*-NL formed an additional conventional hydrogen bond and a π–σ interaction with Trp114, in addition to hydrophobic interactions (Leu73, Leu76, His77, Met89) and van der Waals contacts (Leu80, Gly92, Lys93, Cys95, Leu110), which contributed to enhanced cavity stabilization. These interactions maintained a pocket occupancy pattern comparable to DEET (Figure 3). Moreover, the spatial overlap of binding residues suggests competitive occupation of the same binding pocket [24].

**Figure 4:**
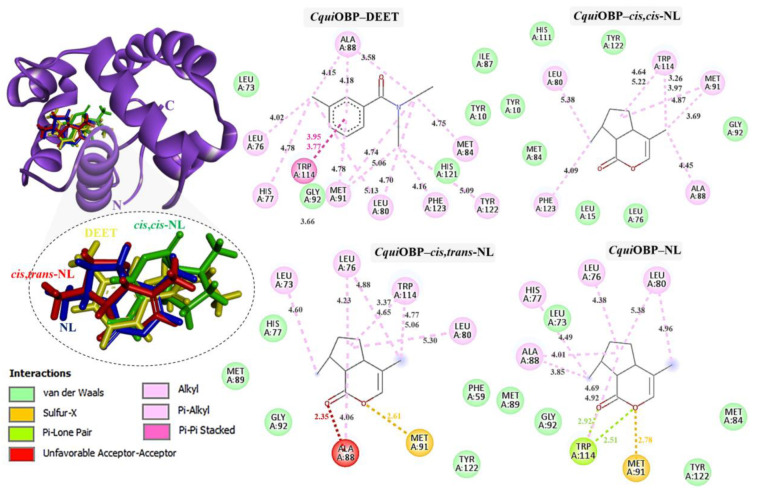
Complexes of *Cqui*OBP with DEET, *cis,cis*-NL, *cis,trans*-NL, and NL are represented in yellow, green, red, and blue sticks, respectively. The dotted circles highlight the magnified superimposition of DEET and nepetalactone ligands after docking. The right panel shows the 2D schematic interaction diagrams of each complex.

**Figure 5:**
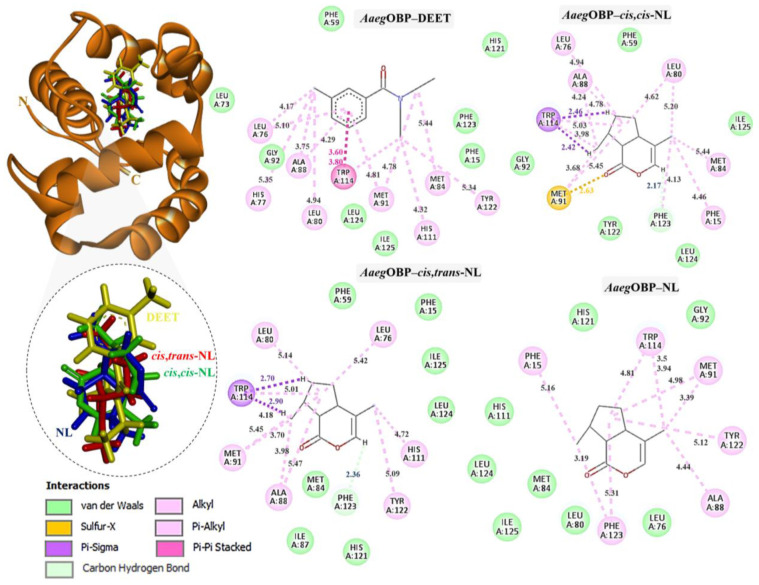
Complexes of *Aaeg*OBP with DEET, *cis,cis*-NL, *cis,trans*-NL, and NL are represented in yellow, green, red, and blue sticks, respectively. The dotted circles highlight the magnified superimposition of DEET and nepetalactone ligands after docking. The right panel shows the 2D schematic interaction diagrams of each complex.

In *Cqui*OBP, DEET bound primarily via hydrophobic interactions with Leu76, His77, Leu80, Ala88, Met91, Tyr122, and Phe123, along with van der Waals interactions with Tyr10, Leu73, Ile87, Gly92, and His121. A crucial π–π stacking interaction with Trp114 was also observed. *Cis,cis*-NL interacted via van der Waals forces (Tyr10, Leu15, Leu76, Gly92, His111) and hydrophobic contacts (Leu80, Ala88, Met91, Trp114, Phe123). *Cis,trans*-NL retained interactions with residues common to DEET (Leu76, Leu80, Trp114) while introducing additional contacts with His77 and Leu73. However, unfavorable steric clashes were detected, including a bump with Ala88 and a sulfur–X interaction with Met91, suggesting possible strain in this binding pose. Nepetalactone showed a strong π–lone pair interaction with Trp114 and a sulfur–X contact with Met91, in addition to van der Waals interactions with Leu73, Met84, Met89, Gly92, and Tyr122. Hydrophobic contacts with His77, Leu80, and Ala88 further supported stable insertion into the binding cavity (Figure 4). For *Aaeg*OBP, DEET engaged a dense hydrophobic network involving Leu76, Ala88, Met91, and Tyr123, closely resembling its binding in *Cqui*OBP. Additional van der Waals interactions were formed with Phe15, Gly92, Phe123, and Leu124. A π–π stacking interaction with Trp114 was again identified as a key stabilizing feature. *Cis,cis*-NL displayed a similar interaction pattern, suggesting that it mimics DEET’s binding mode, with additional π–σ and sulfur–X interactions involving Trp114 and Met91, respectively. *Cis,trans*-NL formed stabilizing contacts with His111, with Trp114 engaging in a key π–σ interaction. Additionally, both *cis,cis*-NL and *cis,trans*-NL complexes exhibited hydrogen bonding with *Cqui*OBP, resulting in greater conformational stability of the complexes. NL exhibited the broadest interaction network, featuring van der Waals contacts (Leu76, Leu80, Gly92, His125) and hydrophobic interactions (Phe15, Ala88, Met91, Trp114, Phe123), which highlights its strong cavity occupancy and stabilization potential (Figure 5).

Thus, these interaction profiles indicate that nepetalactones establish more diverse and stabilizing contacts than DEET, often involving the conserved Trp114 residue. This suggests that nepetalactones may achieve stronger and more specific stabilization within the OBP binding pockets, supporting their potential as effective bio-repellent ligands.

### 2.4. Molecular Dynamics (MD) Simulations Analysis

Molecular dynamics (MD) simulations were performed to evaluate the binding stability and conformational dynamics of DEET, *cis,cis*-NL, *cis,trans*-NL, and NL with the three OBP isoforms. Binding free energy, root-mean-square deviation (RMSD), root-mean-square fluctuation (RMSF), solvent-accessible surface area (SASA), radius of gyration (Rg), and H-bond analysis were assessed to characterize the stability and conformational behavior of the ligand–protein complexes (Table 3) [32,33].

#### 2.4.1. Binding Free Energy (∆G_MM-PBSA_) Calculation

All ligands exhibited favorable binding free energies, confirming their ability to form stable complexes with the OBP receptors. These energy profiles were consistent across duplicate simulation (1st and 2nd runs) (Table 3). DEET, used as the reference ligand, exhibited consistently strong binding energies across both simulation runs with *Agam*OBP (−24.04 and −23.00 kcal/mol), *Cqui*OBP (−22.77 and −23.36 kcal/mol), and *Aaeg*OBP (−22.24 and −23.07 kcal/mol), reaffirming its established role as a broad-spectrum synthetic repellent. Notably, *cis,cis*-NL displayed binding to *Agam*OBP (−24.12 and −24.35 kcal/mol), *Cqui*OBP (−20.66 and 19.96 kcal/mol), and *Aaeg*OBP (−21.45 and −19.05 kcal/mol), revealing *cis,cis*-NL exhibited stronger binding to *Agam*OBP. This suggests that *cis,cis*-NL may act as a competitive or alternative bio-repellent to DEET, particularly in *A. gambiae*. *Cis,trans*-NL exhibited slightly stronger binding to *Aaeg*OBP (−23.05 and −23.83 kcal/mol) than DEET, while its interaction with *Cqui*OBP (−21.14 and −20.85 kcal/mol) and *Agam*OBP (−22.14 and −20.92 kcal/mol) were slightly lower, approaching the binding energy of DEET. NL–*Aaeg*OBP complex exhibited higher binding energy (−23.11 and −24.31 kcal/mol) compared to the DEET complex. The overall similarity in binding energies across the three OBPs indicates conserved ligand–protein recognition, although subtle variations among isoforms point to species-dependent preferences in ligand accommodation. Collectively, the molecular dynamics results indicate that *Cis,trans*-NL and NL exhibit binding stability and favorable interaction energetics comparable to those of DEET, with particularly higher binding affinity observed in *Aaeg*OBP in both simulation replicates.

#### 2.4.2. Root Mean Square Deviation (RMSD)

**Figure 6:**
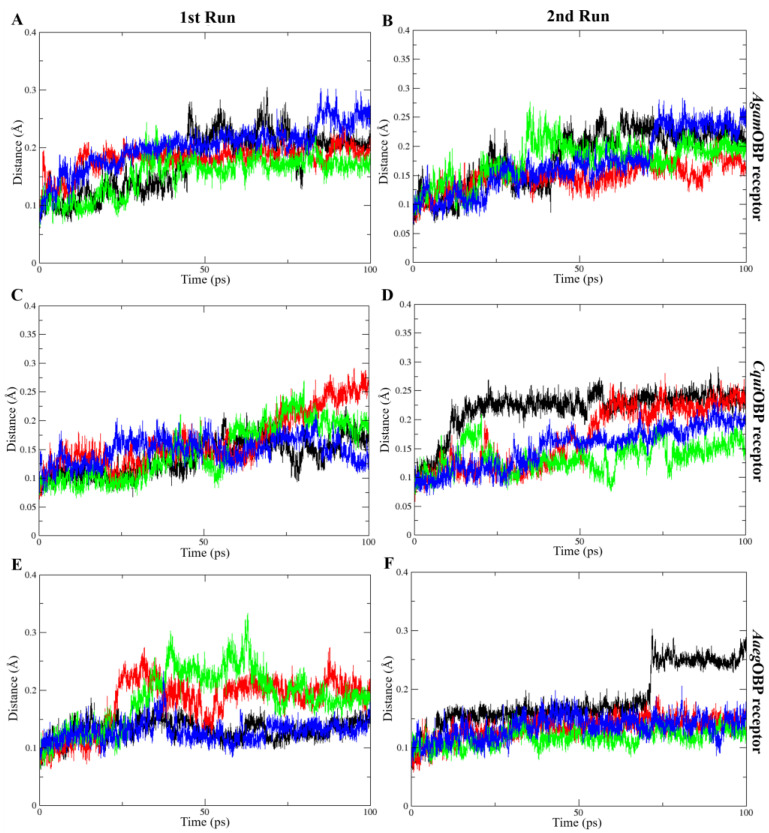
Root-mean-square deviation (RMSD) plots over time for the 1st run (**left panels**) and 2nd run (**right panels**) of *Agam*OBP (**A**,**B**), *Cqui*OBP (**C**,**D**), and *Aaeg*OBP (**E**,**F**) complexes with DEET, *cis,cis*-nepetalactone, *cis,trans*-nepetalactone, and nepetalactone, shown as black, red, green, and blue lines, respectively.

The average RMSD values of all OBP–ligand complexes during molecular dynamics simulations indicated structural stability throughout the trajectories, ranging from 0.13 to 0.20 nm in the 1st run and 0.12 to 0.22 nm in the 2nd run. The average RMSD values of ligands bound to *Agam*OBP showed that the *cis,trans*-NL–*Agam*OBP complex in the 1st run, as well as all ligand–*Agam*OBP complexes in the 2nd run, exhibited lower RMSD values than the DEET complex. Each OBP–ligand complex maintained structural stability throughout the final 10 ns of both simulation trajectories. Similarly, the *Cqui*OBP and *Aaeg*OBP complexes with all ligands, particularly in both runs, exhibited lower RMSD values compared to the DEET complexes. Overall, each complex remained stable during the final 20 ns of both simulations, except for the *cis,cis*-NL–*Cqui*OBP complex, which showed higher RMSD fluctuations before stabilizing in the last 10 ns, and the DEET–*Aaeg*OBP complex, which exhibited elevated RMSD values before reaching stability during the final 30 ns (Figure 6).

#### 2.4.3. Root Mean Square Fluctuation (RMSF)

**Figure 7:**
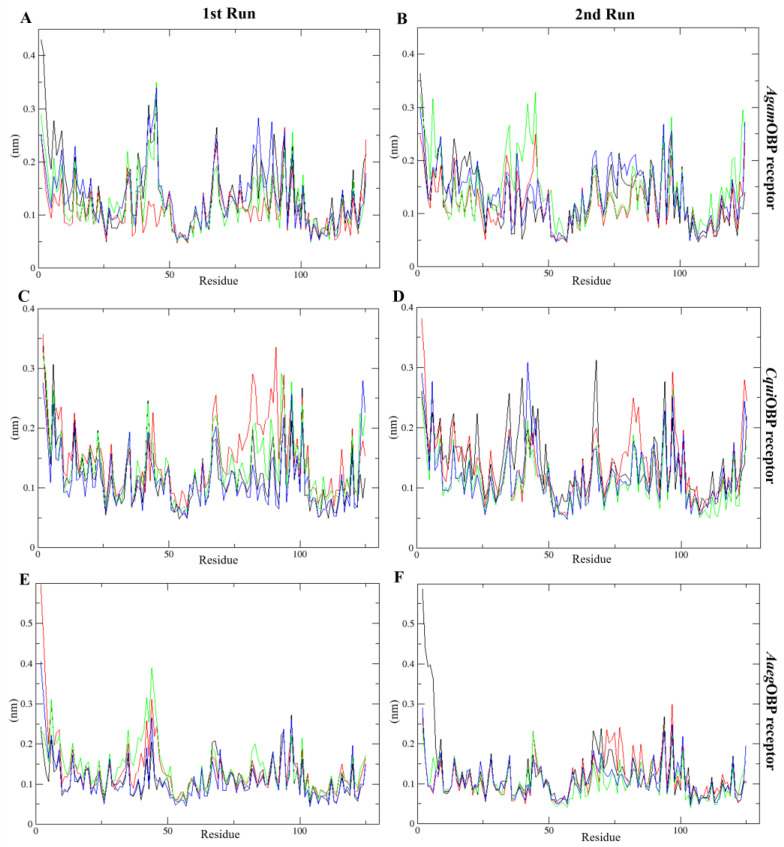
Root-mean-square fluctuation (RMSF) plots over time for the 1st run (**left panels**) and 2nd run (**right panels**) *Agam*OBP (**A**,**B**), *Cqui*OBP (**C**,**D**), and *Aaeg*OBP (**E**,**F**) complexes with DEET, *cis,cis*-nepetalactone, *cis,trans*-nepetalactone, and nepetalactone, represented by black, red, green, and blue lines, respectively.

The RMSF analysis revealed limited residue flexibility, ranging from 0.05 to 0.60 nm and 0.04 to 0.59 nm across all systems in the 1st and 2nd run, respectively. The *Agam*OBP with nepetalactones complexes exhibited narrower backbone fluctuation ranges compared to the *Agam*OBP–DEET complex during both runs. Similarly, *Cqui*OBP bound with *cis,trans*-NL and NL in both simulation replicates showed lower residue fluctuations than the DEET complex. Likewise, *Aaeg*OBP bound with all nepetalactones displayed reduced fluctuations relative to DEET, particularly in 2nd run (Figure 7). However, increased fluctuations were observed for *Aaeg*OBP complexed with DEET in the 2nd run, as well as for all nepetalactone complexes in the 1st run, particularly in the α1 region of the OBP receptor. These observations suggest that although nepetalactones bind effectively, they tend to induce slightly greater local flexibility within the binding pocket, potentially influencing ligand-induced conformational dynamics and odorant recognition. In contrast, DEET binding appears to confer greater rigidity to the protein backbone.

#### 2.4.4. Solvent Accessible Surface Area (SASA) and Radius of Gyration (Rg) Analysis

The solvent-accessible surface area (SASA) values remained stable across all complexes, ranging from 75.02 to 78.87 nm^2^ in the 1st run and 75.00 to 80.29 nm^2^ in the 2nd run. All nepetalactone–OBP complexes exhibited lower SASA values than the DEET complexes, indicating a more compact ligand–protein interface and suggesting stronger encapsulation of the ligand within the binding pocket. These results indicate that DEET binding results in slightly greater protein surface exposure (Table 3). The radius of gyration (Rg) values were also consistent across all systems (1.38–1.40 nm in the 1st run and 1.38–1.41 nm in the 2nd run), confirming that ligand binding did not significantly affect the overall compactness of the OBP structures (Table 3). Collectively, these findings suggest that both DEET and nepetalactones stabilize OBPs without inducing major conformational rearrangements, although subtle differences in local flexibility and solvent exposure distinguish their binding behaviors.

#### 2.4.5. Hydrogen Bond Analysis

**Figure 8:**
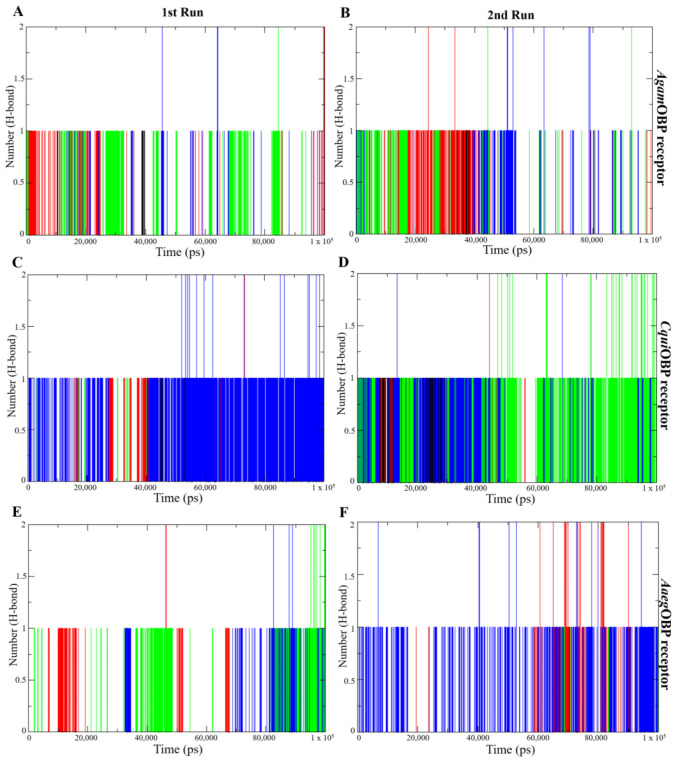
Hydrogen bond (H-bond) analysis plots showing the number of hydrogen bonds over time for the 1st run (**left panels**) and 2nd run (**right panels**) of *Agam*OBP (**A,B**), *Cqui*OBP (**C,D**), and *Aaeg*OBP (**E,F**) complexes with DEET, *cis,cis*-nepetalactone, *cis,trans*-nepetalactone, and nepetalactone, represented by black, red, green, and blue lines, respectively.

Hydrogen bond analysis was performed to assess the stability and strength of nepetalactone binding to the OBP receptors. All nepetalactone–*Agam*OBP complexes consistently maintained one hydrogen bond from the initiation of the simulation up to 50 ns in both runs, and one hydrogen bond beyond 50 ns, with occasional formation of two hydrogen bonds at certain time points. For *Cqui*OBP, the NL complex maintained a single stable hydrogen bond throughout the 1st run and during the first 50 ns of the 2nd run. Similarly, the *cis,trans*-NL–*Cqui*OBP complex exhibited stable hydrogen bonding during the first 50 ns in both runs, whereas the *cis,cis*-NL–*Cqui*OBP complex displayed intermittent hydrogen bonding in the 1st run but demonstrated a more stable single hydrogen bond throughout the 2nd run, with the formation of two hydrogen bonds during the final 50 ns. In contrast, the *Cqui*OBP–DEET complex maintained one hydrogen bond solely within the initial 50 ns of the 1st run. In the case of *Aaeg*OBP, all nepetalactone complexes maintained hydrogen bonds throughout the entire simulation period. Additionally, *cis,cis*-NL–*Aaeg*OBP formed two hydrogen bonds during the last 50 ns of the 2nd run. Notably, the NL–*Aaeg*OBP complex exhibited more consistent formation of a single hydrogen bond throughout the duration of the 2nd run. In contrast, DEET–OBP complexes rarely maintained even a single hydrogen bond and did not exhibit the formation of two hydrogen bonds at any point (Figure 8). These results indicate that nepetalactones form more stable and stronger hydrogen-bonding interactions with OBP receptors compared to DEET.

#### 2.4.6. Interaction Analysis of the Ligands with OBPs Receptors

**Figure 9:**
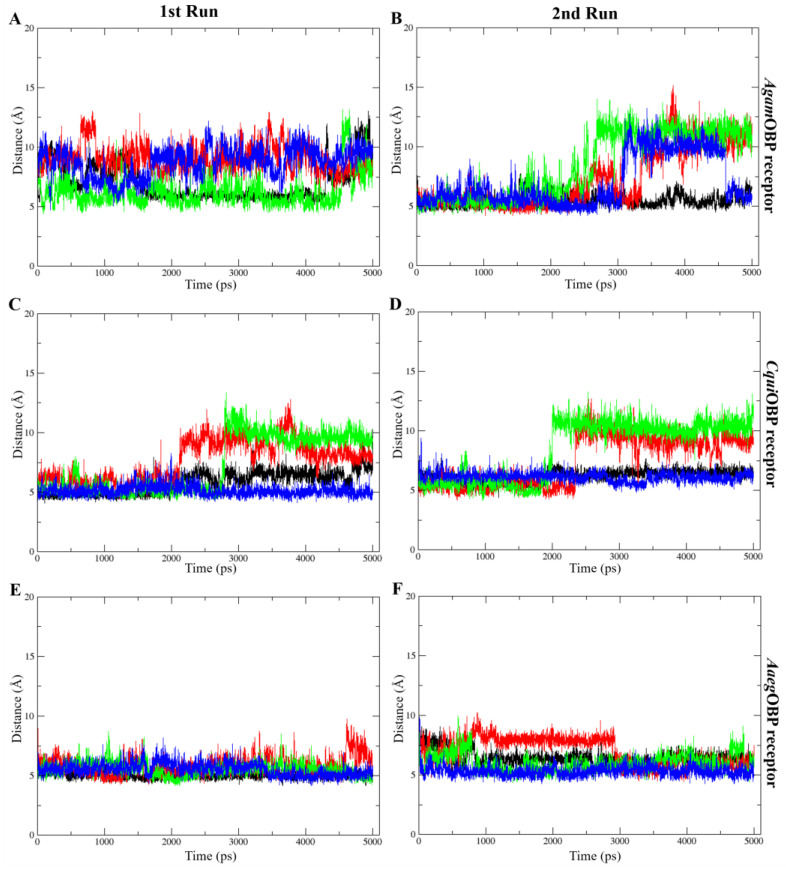
Distance between the center of mass (COM) of each ligand and key amino acids (Trp114) that consistently played a crucial role across all complexes. The 1st run (**left panels**) and 2nd run (**right panels**) are shown for *Agam*OBP (**A**,**B**), *Cqui*OBP (**C**,**D**), and *Aaeg*OBP (**E**,**F**) complexes with DEET, *cis,cis*-nepetalactone, *cis,trans*-nepetalactone, and nepetalactone, represented by black, red, green, and blue lines, respectively.

**Figure 10:**
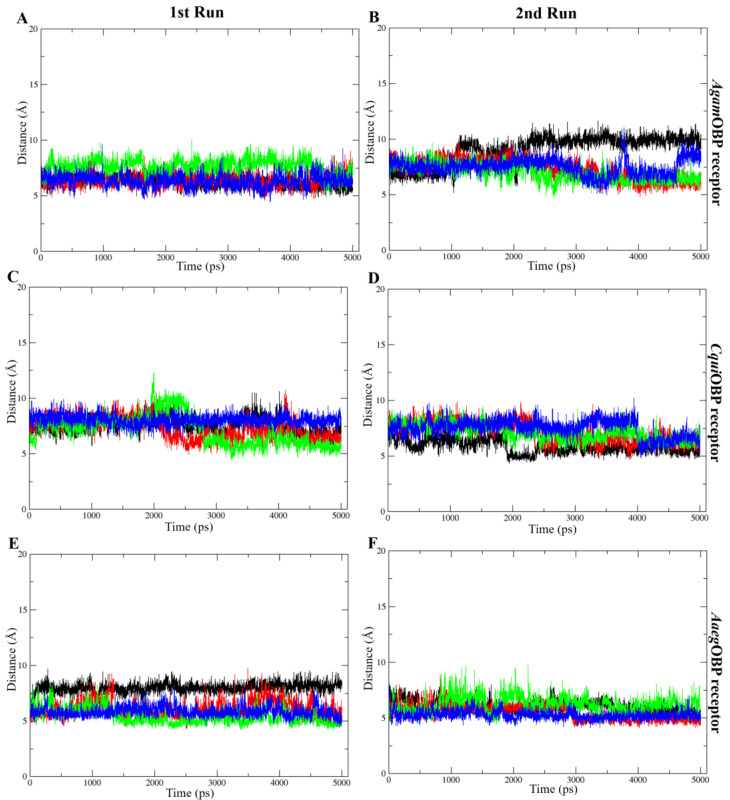
Distance between the center of mass (COM) of each ligand and selected important amino acids, including Leu76 of *Agam*OBP (**A**,**B**) and *Cqui*OBP (**C**,**D**), and Phe123 of *Aaeg*OBP (**E**,**F**). These exhibited the lowest ligand–residue distances throughout the simulation. Panels show the 1st run (**left**) and 2nd run (**right**). All ligands, including DEET, *cis,cis*-nepetalactone, *cis,trans*-nepetalactone, and nepetalactone, are represented by black, red, green, and blue lines, respectively.

The COM distance analysis showed that Trp114 remained the closest and most stable interacting residue across all OBP–ligand complexes (Figure 9). In both simulation runs, all ligands consistently stayed within roughly 4–8 Å of Trp114, indicating sustained proximity throughout the 5 ns trajectories. DEET and nepetalactone exhibited the smallest fluctuations, whereas the nepetalactone isomers showed slightly greater variation in *Agam*OBP and *Cqui*OBP. In contrast, Trp114 in *Aaeg*OBP displayed very low fluctuation with all ligands (Figure 9E,F). These observations support the role of Trp114 as a conserved residue contributing to ligand stabilization in all three OBPs. Similarly, the COM distance profiles indicated that Leu76 (*Agam*OBP and *Cqui*OBP) and Phe123 (*Aaeg*OBP) also maintained consistently short and stable distances to all ligands across both simulation runs (Figure 10). Ligands generally remained within 4–8 Å of these residues, suggesting continuous contact and a likely role in ligand stabilization. Occasional increases in distance were observed for DEET in *Agam*OBP and *Aaeg*OBP, but these did not disrupt the overall trend. Collectively, these residues appear to function as key stabilizing sites within each OBP, maintaining persistent interactions with all ligands throughout the simulations [34,35].

### 2.5. ADMET Analysis

ADMET profiling supported the potential of *cis,cis*-NL, *cis,trans*-NL, and NL as safe, plant-based bio-repellents (Table 4) [36]. *Cis,cis*-NL showed markedly higher water solubility than DEET and other nepetalactones, suggesting enhanced formulation potential. Skin permeability was consistently low, including −2.282, −2.212, and −2.212 log Kp), implying reduced dermal penetration and greater safety compared to DEET. Distribution was minimal, with *cis,cis*-NL showing the highest plasma availability. Moderate BBB penetration was observed, though CNS permeability remained low, minimizing neurological risk. None were CYP3A4 or CYP2D6 substrates; only DEET and *cis,cis*-NL inhibited CYP1A2. Clearance was highest for *cis,cis*-NL, followed by DEET and other isomers. Toxicological predictions indicated low mutagenicity risk. Interestingly, skin sensitization was predicted for DEET and the nepetalactone isomers, but not for *cis,cis*-NL. Overall, *cis,cis*-NL followed by other isoforms demonstrated superior safety, solubility, clearance, and environmental compatibility, highlighting it as the most promising bio-repellent candidate relative to DEET.

## 3. Discussion

The present study integrated phytochemical characterization, molecular docking, molecular dynamics simulations, and ADMET profiling to systematically evaluate nepetalactone and its derivatives from *N. cataria* in comparison with DEET, the widely used active ingredient in commercial insect repellents [5,14]. Despite its efficacy, DEET has been associated with adverse neurological, cardiovascular, dermatological, and gastrointestinal effects, particularly in children, following ingestion or excessive dermal exposure [37]. By employing a multifaceted approach, this study not only elucidated the molecular determinants of repellent activity and the conformational dynamics of mosquito odorant-binding proteins (OBPs) but also assessed the pharmacokinetic and toxicological profiles of nepetalactones. Collectively, these findings provide a strong rationale for the development of nepetalactone-based bio-repellents as safer and more environmentally sustainable alternatives to DEET.

For phytochemical characterization, GC–MS/MS analysis revealed the dominance of iridoid lactones, including *cis,cis*-NL, *cis,trans*-NL, and NL, in extracts obtained via dried leaf oil extraction (DOE) and steam distillation (SDE). In this study, we can identify *cis,trans*-NL (4.89%), *cis,cis*-NL (1.80%), and NL (0.23%) exclusively in the dried leaf oil-based extract. Notably, the SDE fraction included *cis,trans*-NL (52.80%) and *cis,cis*-NL (47.20%) as dominant compositions. These bicyclic iridoid lactones are well established as the key insect-repellent constituents of *N. cataria*. While originally described as feline attractants [38], subsequent studies confirmed their potent repellency against Aedes, Culex, and Anopheles [39,40]. Mechanistically, nepetalactones interact with odorant-binding proteins (OBPs) and olfactory receptors involved in host-seeking, particularly OR2 and OR8, which respond to human kairomones such as octenol and lactic acid [41,42]. We therefore hypothesize that nepetalactones primarily exert their repellent effects by disrupting mosquito olfactory pathways in a manner analogous to DEET’s sensory interference mechanism. Notably, both fresh and dried leaf extracts (FOE and DOE) contained alkanes such as undecane, 2-methyldecahydronaphthalene, and 2,6,10-trimethyldodecane, which likely originated from the olive oil used as the extraction solvent. Although these hydrocarbons are generally less bioactive than iridoids, they may indirectly enhance repellency by influencing formulation volatility and extending the release of active compounds [27,31,43,44]. Structural similarity of branched alkanes to mosquito cuticular hydrocarbons may also create olfactory interference [45]. Thus, hydrocarbons likely act as synergists that stabilize and extend nepetalactone activity. Moreover, FOEs and DOEs were enriched in alcohols (e.g., 2-Hexyl-1-octanol) and esters (e.g., tetrahydrogeranyl formate), which were found in olive oil (Table 1) [46,47]. These moderately volatile compounds can disrupt host-seeking either by masking attractant cues or activating deterrent receptors [48]. Esters, in particular, may function as repellent enhancers. Butylated hydroxytoluene (BHT), the major compound detected in both maceration extracts, likely functions as a natural antioxidant that stabilizes iridoids and may also act as a mild deterrent [49,50]. Dried extracts also contained 1,3-di-tert-butylbenzene (4.70%), an aromatic hydrocarbon with insect-deterrent properties. Interestingly, mixtures of hydrocarbons with iridoids may result in prolonging the effectiveness of long-acting lactone repellents [17,18]. Moreover, these stabilizing agents may not only preserve bioactivity but also contribute synergistically to repellency. Thus, these findings corroborate previous reports indicating that nepetalactone-rich dried extracts possess repellent activity comparable to DEET [14,39]. Notably, DOE maceration represents a simple and eco-friendly extraction approach, potentially enhanced by synergistic contributions from hydrocarbons, oxygenated volatiles, and phenolic compounds. In contrast, SDE yielded the highest concentrations of nepetalactone.

Docking studies demonstrated that all nepetalactone isomers exhibited stronger binding affinities than DEET across the three mosquito OBPs (*Agam*OBP, *Cqui*OBP, and *Aaeg*OBP). A conserved Trp114 residue consistently stabilized ligand binding via π–π and hydrophobic interactions, underscoring its critical role in odorant recognition [24]. Among the isomers, *cis,trans*-NL established the most extensive interaction network, engaging in hydrogen bonding and π–σ interactions with Trp114 in *Agam*OBP, while both *cis,cis*- and *cis,trans*-NL were further stabilized through π–σ interactions with Trp114 of *Aaeg*OBP. Functionally, this is consistent with the known function of OBPs that act as solubilize of hydrophobic odorants and deliver them to olfactory receptors. In contrast, DEET is thought to disrupt this process by binding OBPs with high affinity, thereby preventing recognition of natural attractants [20]. Similarly, nepetalactones form stable hydrophobic and hydrogen-bonding interactions within OBP binding cavities, promoting conformational rigidity [51]. OBPs are characterized by broad ligand specificity, and ligands that engage multiple residues are more likely to alter protein conformational dynamics [52,53]. Such expanded binding footprints may explain the superior binding affinity and potentially enhanced olfactory disruption exhibited by nepetalactones compared to DEET. Additionally, the results suggest that *N. cataria* leaf extract in olive oil may exert an enhanced or synergistic repellent effect, owing to the presence of ligands with binding affinities to mosquito OBPs comparable to or greater than that of DEET. The strong interactions observed for both nepetalactones and these additional ligands reinforce their roles as key bioactive constituents. Collectively, these findings underscore the potential of *N. cataria*-derived compounds, either individually or in combination with carrier oils, as natural alternatives to synthetic repellents.

The molecular dynamics simulations provided critical insights into the conformational behavior of three OBP receptors upon ligand binding, offering mechanistic evidence for the stability and potential efficacy of nepetalactone and derivatives compared to the benchmark repellent DEET. The consistently low RMSD values [54] across all OBP–ligand complexes confirmed that binding did not disrupt the global protein architecture, indicating that nepetalactone isomers are well accommodated within the conserved binding pockets. Such stability is essential for ligand recognition and transport, as OBPs must maintain structural integrity while transiently interacting with semiochemicals [33,55]. Specifically, the *cis,cis*-NL–*Agam*OBP complex demonstrated greater conformational stability and stronger binding affinity than DEET across two independent MD simulations, suggesting that this isomer can form an equally, if not more, stable interaction within the Anopheles gambiae OBP binding pocket. Such stability may reflect more extensive or complementary residue–ligand contacts, consistent with docking predictions that nepetalactones engage broader interaction networks compared to DEET. Equally important is the finding that all nepetalactones binding to *Aaeg*OBP yielded stability comparable to DEET, underscoring the potential cross-species effectiveness of nepetalactones. Since *Aedes aegypti* is a major vector for arboviruses such as dengue, chikungunya, and Zika, the ability of nepetalactones to maintain stable interactions with *Aaeg*OBP receptor is a promising indicator of their applied utility. Furthermore, nepetalactones induced higher RMSFs at key loop regions surrounding the binding pocket. This localized flexibility is critical, as ligand-induced conformational dynamics have been implicated in OBP-mediated odorant release and receptor activation [56,57]. Interestingly, *cis,cis*-NL–*Agam*OBP complexes exhibited reduced SASA, suggesting a more encapsulated ligand conformation. Such tight binding could reduce the accessibility of competing host odorants, thereby enhancing repellent efficacy. By contrast, *cis,trans*-NL promoted slightly higher flexibility in *Aaeg*OBP, suggesting potential differences in species-specific repellency efficacy. For the ligand interaction analysis, COM between the OBPs and all ligands revealed that the key residue is Trp114, contributing to ligand stabilization in all three OBPs. Moreover, Leu76 (*Agam*OBP, *Cqui*OBP) and Phe123 (*Aaeg*OBP) stay closest to the ligand COM during the simulation, indicating stable and consistent interaction. Importantly, nepetalactone isomers with *Agam*OBP and *Aaeg*OBP complexes trend more stable and lower distances compared to DEET. Overall, the results confirm receptor-specific key residues that help stabilize each ligand in the binding pocket. These findings resonate with behavioral studies showing that nepetalactone isomers vary in repellency strength depending on the mosquito species targeted. Thus, nepetalactones appear to exploit structural adaptability, inducing conformational shifts that may interfere with the precise odorant recognition and transport essential for host-seeking behavior. This dual mechanism, effective binding combined with dynamic disruption, represents a novel pathway for repellent function.

ADMET predictions indicated that nepetalactones possess favorable pharmacokinetic and safety profiles compared to DEET. Importantly, all nepetalactones have low skin permeability, indicating greater safety compared to DEET. None of the ligands were predicted as substrates or inhibitors of major CYP450 isoforms (except for CYP1A2 inhibition by DEET and *cis,cis*-NL), suggesting a lower risk of metabolic interference. From a toxicological perspective, DEET was associated with skin sensitization and neurological concerns [58,59]. In contrast, *cis,cis*-NL showed a more benign profile, lacking skin sensitization alerts and hepatotoxicity predictions, while retaining strong binding to mosquito OBPs. Overall, the in silico evidence supports its safer use profile. The combined evidence suggests that nepetalactone isomers represent strong candidates for bio-repellent development. Their ability to bind deeply and flexibly to OBPs, combined with favorable ADMET properties, provides a rational basis for their efficacy and safety compared to DEET.

Importantly, this study represents a substantive advancement over previous nepetalactone–OBP investigations by introducing a comprehensive, multi-target and multi-species computational framework that integrates molecular docking, replicated molecular dynamics simulations, MM-PBSA binding free energy estimation, and binding cavity architecture analysis. Additionally, our approach provides dynamic, quantitative, and mechanistic insights into ligand stability, receptor selectivity, and binding energetics across multiple mosquito species.

Moreover, the plant-derived origin of nepetalactones supports ecological sustainability and aligns with the growing demand for natural and environmentally compatible repellents. Although this work is primarily computational, its major strength lies in the integrated in silico screening of biological activity, binding mechanisms, and safety profiles, enabling rational prioritization of lead compounds for experimental validation. Bioassays and formulation-based safety evaluations are currently underway to substantiate these findings.

## 4. Materials and Methods

### 4.1. Preparation of Nepeta cataria Leaf Extraction

*Nepeta cataria* seeds were imported from the United States of America (Hudson Valley Seed Company, Accord, NY, USA) and were certified by the United States Department of Agriculture (USDA). The plant species was identified and confirmed by a qualified plant taxonomist. For the experiments reported in the manuscript, fresh leaves of *N. cataria* were harvested from an open field in Sai Noi Sub-district, Sai Noi District, Nonthaburi Province, Thailand (coordinates: 13°58’17.2” N latitude, 100°20’16.0” E longitude). Nepetalactones were extracted from catnip leaves by using steam distillation. A total of 100 g of dried leaf was suspended in 2.5 L of distilled water and subjected to distillation in a stainless-steel apparatus for 8 h, boiling. The hydrosol fraction was collected and stored in a tightly sealed container at 4 °C for further use [60,61,62]. For extraction by the maceration method using olive oil solvent, both fresh and dried catnip leaves were used. For dried samples, leaves were cut into small pieces and dried in a hot-air oven at 45 °C for 8 h to remove moisture. Fresh leaves were cut into small pieces without prior drying. Each sample was macerated in olive oil at a 1:10 (*w*/*v*) ratio (sample: olive oil), using the same raw material weight for both fresh and dried samples. The mixtures were incubated at 90 °C for 2 h with intermittent stirring to facilitate extraction. The extracts were filtered through a muslin cloth (100–400 mesh; approximate pore size 37–149 µm) to remove coarse plant debris. The filtrates were collected and stored at 4 °C in a tightly sealed container until further analysis.

### 4.2. Identification of Chemical Compositions by Using GC-MS/MS

After extraction, 4 mL of catnip extract was mixed with 2 mL of hexane. Then, 0.4 mL of 0.9% NaCl solution and anhydrous MgSO_4_ powder were added to remove residual water from the sample. From the mixture, the hexane layer (upper phase) was transferred into a clean vial and evaporated under a gentle stream of nitrogen gas. The concentrated residue was re-dissolved in 1 mL of diethyl ether. A 2 μL aliquot of the prepared sample was injected into the GC–MS/MS system. The analysis was performed using a Trace 1610 system equipped with a TG-5MS capillary column (30 m × 0.25 mm i.d., 0.25 μm film thickness, Thermo Scientific, United States). Helium was used as the carrier gas at a constant flow rate of 1 mL/min. The oven temperature program was set to an initial temperature of 70 °C, increased at 2 °C/min to 170 °C, and then further increased at 5 °C/min to 230 °C. Electron impact (EI) ionization was employed at 50 eV. The total run time was 63 min. A TSQ 9610 Triple quadrupole mass spectrometer (Thermo Scientific, Waltham, MA, USA) was used as a detector. The mass spectra were identified by comparison with the NIST 2020 mass spectral library (Gaithersburg, MD, USA), and compounds from the catnip leaf extract that showed a match of more than 70% with the library were selected [62].

### 4.3. Structural Analysis and Comparison of AgamOBP, CquiOBP, and AaegOBP

The experimental protein models analyzed were Odorant-Binding Proteins (OBPs). Three OBPs were selected, including *Cqui*OBP1 from *Culex quinquefasciatus* (PDB ID: 3OGN), *Aaeg*OBP1 from *Aedes aegypti* (PDB ID: 3K1E, chain A), and *Agam*OBP1 from *Anopheles gambiae* (PDB ID: 3N7H) [20,63,64]. The 3D structures were retrieved in PDB format from the RCSB Protein Data Bank (https://www.rcsb.org/, accessed on 30 June 2025) and were virtualized by using Discovery Studio (version 2024). All OBPs sequences were aligned on Clustal Omega (version 1.2.4) (https://www.ebi.ac.uk/jdispatcher/msa/clustalo?stype=protein, accessed on 1 July 2025). The conserved amino acids were generated with Multiple Align Show (https://www.bioinformatics.org/sms/multi_align.html, accessed on 1 July 2025).

### 4.4. Molecular Docking of N. Cataria Phytochemicals with the OBP Receptors

All 3D structures of OBPs and the selected ligands used, with their SDF structures obtained from PubChem (https://pubchem.ncbi.nlm.nih.gov/, accessed on 2 July 2025). Molecular docking was performed using CB-Dock2 (Cavity-detection guided Blind Docking) server (https://cadd.labshare.cn/cb-dock2/index.php, accessed on 5 July 2025), which provided binding cavity parameters (Å) [30]. This method employs a blind docking approach using AutoDock Vina as the docking engine. Docking scores represent Vina-estimated binding affinities (kcal/mol). Each ligand–protein complex was docked in triplicate, and the pose with the lowest docking score was selected for further analysis. After that, the resulting protein–ligand complexes were visualized and analyzed with Discovery Studio (version 2024).

### 4.5. Molecular Dynamics (MD) Simulations 

Molecular dynamics (MD) simulations were performed to investigate the interactions between *Agam*OBP, *Cqui*OBP, and *Aaeg*OBP receptors and three nepetalactone isoforms as ligands. All simulations were conducted using GROMACS 5.1.4 with the OPLS/AA (Optimized Potentials for Liquid Simulations-All Atom) force field, a well-established choice for biomolecular simulations due to its balance of accuracy and efficiency [65]. Each system was solvated in an orthorhombic box filled with TIP3P (Transferable Intermolecular Potential with 3 Points) water molecules, a widely used model that is fully compatible with the OPLS/AA force field. Counterions (Na^+^ and Cl^−^) were added to neutralize the system and achieve a physiological salt concentration of 0.15 M. Energy minimization was followed by equilibration under NVT and NPT ensembles. Temperature was maintained at 300 K using the Nosé–Hoover thermostat, while pressure was controlled at 1.0 bar with the Martyna–Tobias–Klein barostat. Production simulations were conducted for 100 ns, a timescale commonly used in protein–ligand molecular dynamics studies, which is sufficient to capture system equilibration and to assess binding stability [66,67]. In this condition, the force field, solvation model, box type, and thermostat/barostat reflect widely accepted best practices in the field, ensuring proper system equilibration and a reliable assessment of protein–ligand interactions [65,67]. Each of the ligand–OBP complexes was subjected to molecular dynamics (MD) simulations in duplicate to validate the results and ensure reproducibility [68].

Trajectory analyses included the calculation of structural parameters, such as root mean square deviation (RMSD), root mean square fluctuation (RMSF), solvent-accessible surface area values (SASA), gyration radius (Gy), and hydrogen bond interactions. Binding free energies (Δ*G_MM-PBSA_*) were estimated using the g_mmpbsa tool, which applies the molecular mechanics Poisson–Boltzmann surface area (MM-PBSA) method within GROMACS [69,70]. Graphical analyses were performed using QtGrace (version 0.2.6).

According to the definition of the MM-PBSA method, binding free energy (Δ*G_bind_*) can be defined by the following equations:

$$∆G_{MM-PBSA}=\;∆G_{bind}=\;G_{complex}-(G_{protein}+G_{ligand})\;$$

where *G_complex_*, *G_protein,_* and *G_ligand_* represent the free energies of the complex, protein, and ligand, respectively. In addition, the binding free energy (Δ*G_bind_*) consists of two parts:

$$∆G_{bind}=\;∆G_{gas}+∆G_{sol}$$

where total gas phase energy (∆*G_gas_*) consists of the electrostatic energy (∆*G_ele_*) and the van der Waals energy (∆*G_vdW_*), which are significant for binding. Total solvation energy (∆*G_sol_*) consists of non-polar solvation energy (∆*G_np_*) and polar solvation energy (∆*G_pb_*).

### 4.6. Pharmacokinetic Evaluation Using Computational ADMET Analysis

To evaluate safety, the pharmacokinetics and toxicity of nepetalactone and its isomers were compared to those of DEET using the pkCSM pharmacokinetics server (https://biosig.lab.uq.edu.au/pkcsm/prediction, accessed on 29 June 2025). Canonical SMILES for each compound were obtained from PubChem and submitted for ADMET (Absorption, Distribution, Metabolism, Excretion, and Toxicity) analysis. Predicted parameters included solubility, P-glycoprotein interactions, and skin permeability. Distribution properties assessed volume of distribution, plasma protein binding, blood–brain barrier penetration, and CNS access. Metabolism was evaluated through cytochrome P450 inhibition and substrate predictions. Excretion was estimated via renal clearance and OCT2 substrate status. Toxicity profiles included human and animal systemic toxicity, hepatotoxicity, skin sensitization, cardiotoxicity, and ecotoxicity [71]. This computational assessment was applied since repellents, though topically used, can penetrate skin and cause systemic or local toxicity, as reported for DEET [24].

## 5. Conclusions

This study integrated phytochemical analysis, molecular docking, molecular dynamics simulations, and ADMET profiling to comprehensively evaluate nepetalactone isomers from *N. cataria* in comparison with DEET. GC–MS/MS confirmed nepetalactones as dominant constituents in steam distillation extraction (SDE) followed by dried leaf maceration extract (DOE), corroborating their established role as key insect-repellent compounds. Additional constituents, including hydrocarbons, alcohols, esters, and antioxidants present in maceration extracts, may synergistically enhance repellent efficacy and stability. Docking and dynamics simulations revealed that nepetalactones exhibit stronger and more extensive interactions with mosquito OBPs than DEET, particularly through conserved residues such as Trp114. These interactions were accompanied by favorable conformational stability and localized flexibility, suggesting dual mechanisms of action: strong binding and disruption of odorant recognition. ADMET predictions further highlighted the safety advantages of nepetalactones, contrasting with DEET’s reported neurological and dermatological concerns. Collectively, these findings underscore the potential of nepetalactone-based formulations as safe, effective, and environmentally sustainable alternatives to synthetic repellents. In further study, we will combine electrophysiological validation with behavioral bioassays across mosquito species, alongside optimized formulation strategies to balance volatility and long-term stability for practical field applications.

## Figures and Tables

**Table 1:** Compound composition of *N. cataria* identified by GC–MS/MS from stream distillation (SDE) and from fresh (FOE), dried leaf oil extractions (DOE), and olive oil.

Compound	Formula	RT (min)	% Peak Area			
			SDE	FOE	DOE	Olive Oil
Polycyclic Alkane						
*trans*-4a-Methyl-decahydronaphthalene	C_11_H_20_	10.48	0	0.84	0.27	1.16
2-Methyldecahydronaphthalene	C_11_H_20_	11.18	0	1.27	0.69	1.31
1,6-Dimethyldecahydronaphthalene	C_12_H_22_	12.97	0	0	0.45	0
Cycloalkane						
*trans*-1,4-Dimethylcyclooctane	C_10_H_20_	9.04	0	0	0	6.86
Pentylcyclohexane	C_11_H_22_	11.5	0	1.45	0.39	1.29
1,4-Dimethyl-2-octadecylcyclohexane	C_26_H_54_	46.92	0	0	0.59	0
Alkene						
2,4-Dimethyl-1-heptene	C_9_H_18_	3.14	0	14.18	4.36	22.2
(2*Z*)-7-Methyl-2-decene	C_11_H_22_	7.04	0	0	0.33	1.35
(3*E*)-3-Heptadecene	C_17_H_34_	41.73	0	0	1.50	0
1-Tetracosene	C_24_H_48_	45.9	0	0	0.33	0
1-Hexacosene	C_26_H_52_	46.51	0	0	0.77	0
Alcohol						
3,7-Dimethyl-1-octanol	C_10_H_22_O	9.13	0	4.11	1.73	0
2-Butyloctanol	C_12_H_26_O	20.58	0	3.02	1.78	3.08
2-Isopropyl-5-methyl-1-heptanol	C_11_H_24_O	21.08	0	3.49	2.51	4.05
11-Methyldodecanol	C_13_H_26_O	21.43	0	0	0	3.01
2-Methyl-1-decanol	C_11_H_24_O	21.55	0	2.56	1.90	0
3,7,11-Trimethyl-1-dodecanol	C_15_H_32_O	33.21	0	0	0.56	0
2-Hexyldecanol	C_16_H_34_O	33.77	0	0	0.60	0.91
2-Hexyl-1-octanol	C_14_H_30_O	34.78	0	1.49	0.94	0.87
2-Octyl-1-dodecanol	C_20_H_42_O	35.35	0	0	0.71	0
2-Hexyldodecanol	C_18_H_38_O	44.99	0	0	0.40	0
2-*cis*-9-Octadecenyloxyethanol	C_20_H_40_O_2_	55.74	0	0	0.65	0
Ester						
Tetrahydrogeranyl formate	C_11_H_20_O_2_	9.30	0	4.48	1.94	7.57
Carbonic acid, eicosyl vinyl ester	C_23_H_44_O_3_	48.23	0	0	0.60	0
Glycidyl (Z)-9-Heptadecenoate	C_20_H_36_O_3_	60.62	0	0	0.87	0
Glycidyl palmitate	C_19_H_36_O_3_	62.54	0	0	10.77	0
9-Octadecenoic acid (Z)-, oxiranylmethyl ester	C_21_H_38_O_3_	62.72	0	0	5.45	0
Sulfonamide						
N-(2-Hydroxyethyl)-N-methyl-perfluorobutane-1-sulfonamide	C_7_H_8_F_9_NO_3_S	15.85	0	1.87	0	0
Aromatic Hydrocarbon						
1,3-Di-tert-butylbenzene	C_14_H_22_	17.63	0	0	4.71	0
Lactone (Iridoid)						
*cis-cis*-Nepetalactone	C_10_H_14_O_2_	23.58	52.80	0	4.90	0
*cis,trans*-Nepetalactone	C_10_H_14_O_2_	25.21	47.20	0	1.80	0
Nepetalactone	C_10_H_14_O_2_	25.58	0	0	0.24	0
Sesquiterpene						
Caryophyllene	C_15_H_24_	26.99	0	0	0.12	0
Aromatic Ketone						
4-Methoxy-3-(isopenten-2-yl)acetophenone	C_13_H_16_O_2_	30.52	0	2.05	1.35	0
Butylated Hydroxytoluene	C_15_H_24_O	32.50	0	31.79	18.22	4.81
Unsaturated Fatty Acid						
6-Octadecenoic acid	C_18_H_34_O_2_	58.47	0	0	0.61	0
Oleic Acid	C_18_H_34_O_2_	59.97	0	0	0.75	0
9-Octadecenoic acid	C_18_H_34_O_2_	61	0	0	0.49	0
(*E*)-13-Docosenoic acid	C_22_H_42_O_2_	61.48	0	0	0.49	0
*cis*-13-Eicosenoic acid	C_20_H_38_O_2_	61.66	0	0	1.03	0
*cis*-11-Eicosenoic acid	C_20_H_38_O_2_	62.14	0	0	0.83	0
Alkane group						
		3.43 to 54.91	0	26.97	22.80	41.53

**Table 2:** Molecular docking of nepetalactones and DEET ligands with *Agam*OBP, *Cqui*OBP, and *Aaeg*OBP receptors. The lowest binding energy (kJ/mol) in terms of Vina scores and putative cavity size obtained from CB-Dock2 server.

OBPs	Cavity Size (Å^3^)	DEET	*cis,cis*-NL	*Cis,trans*-NL	NL
*Agam*OBP	1159	−6.4	−6.6	−6.6	−6.6
*Cqui*OBP	1643	−6.3	−6.6	−6.7	−6.5
*Aaeg*OBP	1284	−6.2	−7.0	−6.7	−7.0

**Table 3:** Analysis of binding free energy (∆*G_MM-PBSA_*), RMSD, RMSF, SASA, and radius of gyration of OBP–ligand complexes after a 100 ns simulation.

OBPs	Ligands	Δ*G_MM-PBSA_* (kcal/mol)	RMSD (nm)	RMSF (nm)	SASA (nm^2^)	Gyration (nm)
*Agam*OBP	DEET (1st run)	−24.04	0.17	0.43–0.05	78.87	1.40
	DEET (2nd run)	−23.00	0.18	0.36–0.05	77.16	1.40
	*cis,cis*-NL (1st run)	−24.12	0.18	0.26–0.05	75.02	1.38
	*cis,cis*-NL (2nd run)	−24.35	0.15	0.26–0.04	77.16	1.39
	*cis-trans*-NL (1st run)	−22.14	0.15	0.35–0.05	77.76	1.40
	*cis-trans*-NL (2nd run)	−20.92	0.17	0.33–0.05	77.09	1.41
	NL (1st run)	−22.72	0.20	0.34–0.05	76.13	1.39
	NL (2nd run)	−23.50	0.17	0.30–0.05	76.67	1.39
*Cqui*OBP	DEET (1st run)	−22.77	0.14	0.34–0.05	77.68	1.39
	DEET (2nd run)	−23.36	0.22	0.31–0.06	78.57	1.40
	*cis,cis*-NL (1st run)	−20.66	0.17	0.36–0.06	77.24	1.39
	*cis,cis*-NL (2nd run)	−19.96	0.17	0.38–0.05	77.16	1.39
	*cis-trans*-NL (1st run)	−21.14	0.15	0.32–0.06	76.62	1.40
	*cis-trans*-NL (2nd run)	−20.85	0.13	0.27–0.05	76.49	1.39
	NL (1st run)	−20.56	0.15	0.28–0.05	76.78	1.39
	NL (2nd run)	−21.91	0.15	0.30–0.05	77.31	1.39
*Aaeg*OBP	DEET (1st run)	−22.24	0.13	0.27–0.05	77.00	1.39
	DEET (2nd run)	−23.07	0.19	0.59–0.06	75.71	1.38
	*cis,cis*-NL (1st run)	−20.45	0.18	0.60–0.06	76.98	1.39
	*cis,cis*-NL (2nd run)	−19.05	0.13	0.30–0.05	75.00	1.38
	*cis-trans*-NL (1st run)	−23.05	0.19	0.40–0.05	78.13	1.40
	*cis-trans*-NL (2nd run)	−23.83	0.12	0.24–0.04	75.10	1.39
	NL (1st run)	−23.11	0.13	0.41–0.05	76.61	1.39
	NL (2nd run)	−24.31	0.13	0.29–0.05	75.67	1.38

**Table 4:** The ADMET properties of *cis,cis*-NL, *cis,trans*-NL, and NL compared to DEET using pkCSM pharmacokinetics server.

Property	Model Name	Predicted Value	*cis,cis*-NL	*cis,trans*-NL	NL	Unit
Absorption	Water solubility	−2.65	−0.10	−2.52	−2.52	Numeric (log mol/L)
Absorption	Skin Permeability	−2.67	−2.28	−2.21	−2.21	Numeric (log Kp)
Absorption	P-glycoprotein substrate	No	No	No	No	Categorical (Yes/No)
Absorption	P-glycoprotein I & II inhibitor	No	No	No	No	Categorical (Yes/No)
Distribution	VDss (human)	0.16	0.28	0.19	0.19	Numeric (log L/kg)
Distribution	BBB permeability	0.36	0.29	0.70	0.70	Numeric (log BB)
Distribution	CNS permeability	−1.93	−2.83	−2.08	−2.08	Numeric (log PS)
Metabolism	CYP2D6 and CYP3A4 substrate	No	No	No	No	Categorical (Yes/No)
Metabolism	CYP1A2 inhibitor	Yes	Yes	No	No	Categorical (Yes/No)
Metabolism	CYP2C9, CYP2D6 and CYP3A4 inhibitor	No	No	No	No	Categorical (Yes/No)
Excretion	Total Clearance	0.59	0.91	0.11	0.11	Numeric (log mL/min/kg)
Excretion	Renal OCT2 substrate	No	No	No	No	Categorical (Yes/No)
Toxicity	hERG I & II inhibitor	No	No	No	No	Categorical (Yes/No)
Toxicity	Oral Rat Acute Toxicity (LD_50_)	2.31	2.48	1.77	1.77	Numeric (mol/kg)
Toxicity	Oral Rat Chronic Toxicity (LOAEL)	1.46	4.57	2.30	2.30	Numeric (log mg/kg_bw/day)
Toxicity	Hepatotoxicity	No	No	No	No	Categorical (Yes/No)
Toxicity	Skin Sensitisation	Yes	No	Yes	Yes	Categorical (Yes/No)
Toxicity	*T.Pyriformis* toxicity	0.59	−0.96	0.23	0.23	Numeric (log µg/L)
Toxicity	Minnow toxicity	1.19	4.81	1.30	1.30	Numeric (log mM)

## Data Availability

The original contributions presented in this study are included in this article. Further inquiries can be directed to the corresponding author.

## Supplementary Materials

The following supporting information can be downloaded at: https://www.mdpi.com/article/10.3390/ijms27031572/s1.

## Abbreviations

The following abbreviations are used in this manuscript:

NL	Nepetalactones
*Cis,cis*-NL	*Cis,cis*-Nepetalactones
*Cis,trans*-NL	*Cis,trans*-Nepetalactones
DEET	N,N-diethyl-meta-toluamide
SDE	Stream distillation
FOE	Fresh leaf in olive oil extraction
DOE	Dried leaf in olive oil extraction
OBPs	Odorant-binding proteins
PDB	Protein data bank
RMSD	Root mean square deviation
RMSF	Root mean square fluctuation
Gy	Gyration radius
SASA	Solvent-accessible surface area values
