# Supplementary material for: A Safer Alternative Bio-Repellent: Targeting Mosquito Odorant-Binding Proteins with Catnip-Derived Nepetalactones from Nepeta cataria Leaves
Source: Int J Mol Sci. 2026 Feb 5;27(3):1572. doi: 10.3390/ijms27031572 (PMC12897829; doi:10.3390/ijms27031572)
Supplement: Supplementary file 1 [file ijms-27-01572-s001.zip › ijms-4093069-supplementary.pdf]

International Journal of
Molecular Sciences

MDPI

Article

A Safer Alternative Bio-Repellent: Targeting Mosquito Odorant-Binding Proteins with Catnip-Derived Nepetalactones from *Nepeta cataria* Leaves

Tarawin Kiatlertpongsa [1,2], Siriporn Nonkhwao [1,3], Jarupa Charoenrit [1,3], Jirawat Saetan [4], Supawadee Duangprom [1,3], Sineenart Songkoomkrong [1,3], Prateep Amonruttanapun [1,3], Piyapon Janpan [1,3], Prasert Sobhon [5], Sakda Daduang [6] and Napamanee Kornthong [1,3,*]

1 Research Unit in Innovative Marine Biotechnology and Natural Bio-Resources for Sustainable Health and Wellness, Thammasat University, Pathumthani 12120, Thailand; kiatlertpongsa@gmail.com (T.K.); siriphorn.nonkhaow@gmail.com (S.N.); jaa.charupa@gmail.com (J.C.); su.duangprom@gmail.com (S.D.); sineenartsong@gmail.com (S.S.); wanderer_sci@yahoo.com (P.A.); piyapon.ater@gmail.com (P.J.)
2 Ruamrudee International School, Minburi Campus, Bangkok 10510, Thailand
3 Chulabhorn International College of Medicine, Thammasat University, Rangsit Campus, Pathumthani 12120, Thailand
4 Division of Health and Applied Sciences, Faculty of Science, Prince of Songkla University, Hat Yai 90110, Songkhla, Thailand; jisaetan@gmail.com
5 Department of Anatomy, Faculty of Science, Mahidol University, Bangkok 10400, Thailand; prasert.sob@mahidol.ac.th
6 Division of Pharmacognosy and Toxicology, Faculty of Pharmaceutical Sciences, Khon Kaen University, Khon Kaen 40002, Thailand; sakdad@kku.ac.th
* Correspondence: napamaneenatt@gmail.com or napanatt@tu.ac.th

 https://doi.org/10.3390/ijms27031572

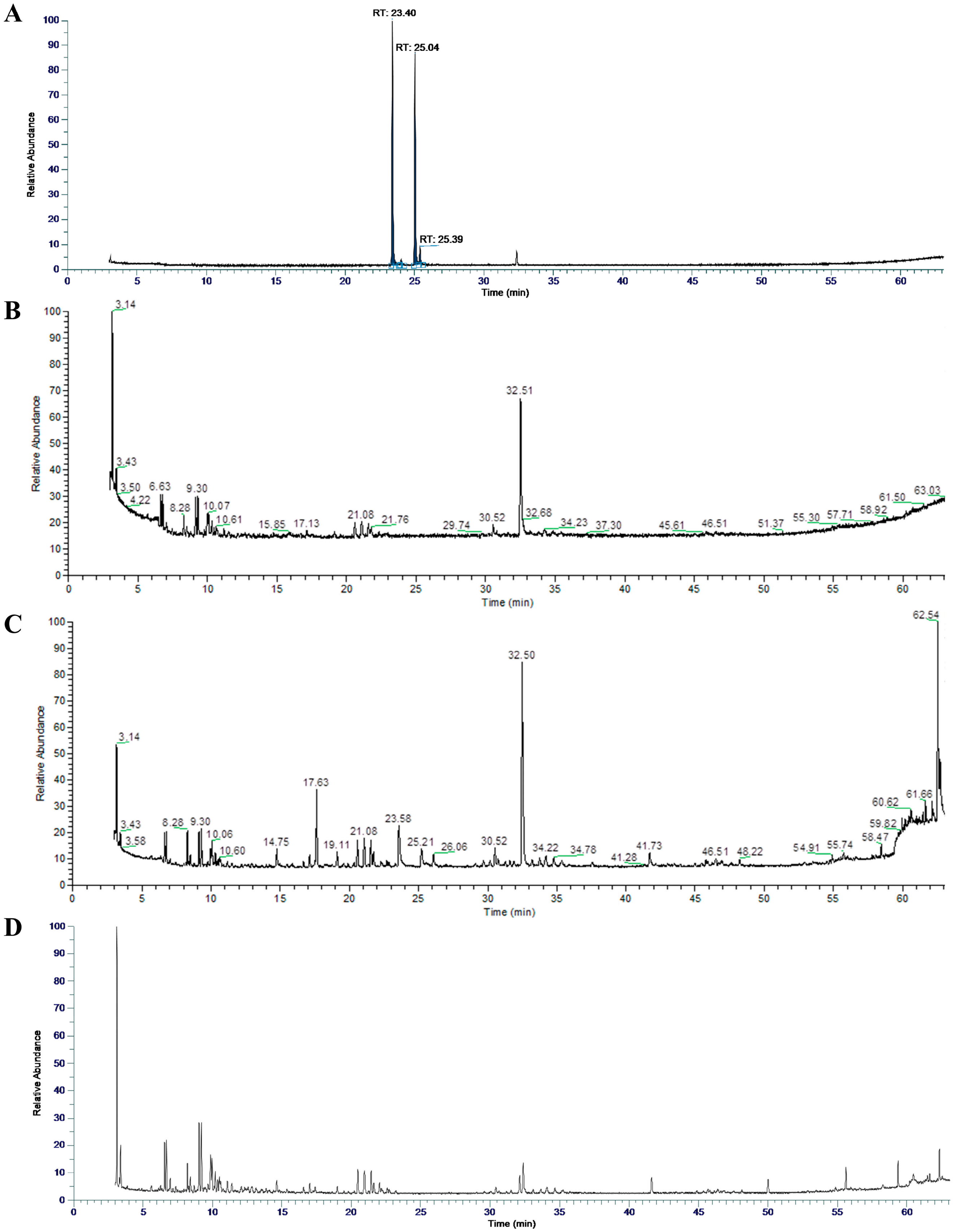

Figure S1. GC–MS/MS chromatograms of *N. cataria* leaf extracts obtained using different methods: (**A**) steam distillation (SDE), (**B**) fresh leaf olive oil extraction (FOE), (**C**) dried leaf olive oil extraction (DOE), and (**D**) olive oil.

https://doi.org/10.3390/ijms27031572

Table S1. Molecular docking was performed with common ligands identified across all fractions, including fresh (FOE) and dried (DOE) leaf oil extracts, as well as olive oil, against the *Agam*OBP, *Cqui*OBP, and *Aaeg*OBP receptors. The lowest binding energies (kJ/mol), based on Vina scores, along with the putative cavity sizes, were obtained using the CB-Dock2 server.

Ligand	Group	*Agam*OBP	*Cqui*OBP	*Aaeg*OBP
2,4,6-Trimethyldecane	Alkane	−6.2	−6.7	−6.3
2,4-Dimethyl-1-heptene	Alkene	−5.4	−5.6	−5.0
2,6,10-Trimethyldodecane	Alkane	***−6.6***	***−6.9***	***−7.0***
2,6-Dimethylnonane	Alkane	−5.7	−6.4	−5.3
2-Butyloctanol	Alcohol	−6.2	−6.2	−5.8
2-Hexyl-1-octanol	Alcohol	−6.3	−6.8	−6.0
2-Isopropyl-5-methyl-1-heptanol	Alcohol	−6.0	−4.3	−5.6
2-Methyldecahydronaphthalene	Polycyclic Alkane	***−6.9***	***−7.2***	***−6.7***
2-Methyldecane	Alkane	−5.7	−5.9	−5.5
3,3-Dimethyloctane	Alkane	−5.6	−6.1	−5.1
4,6-Dimethyldodecane	Alkane	-6.6	−6.7	−6.3
4,7-Dimethylundecane	Alkane	−6.1	−6.7	−5.9
4-Methyloctane	Alkane	−5.3	−5.6	−5.0
4-Methylundecane	Alkane	−5.6	−6.2	−5.7
Butylated Hydroxytoluene	Aromatic Ketone	***−7.1***	***−6.6***	***−7.7***
Pentylcyclohexane	Cycloalkane	−6.3	−6.6	−6.1
Tetrahydrogeranyl formate	Ester	−5.7	−5.8	−5.9
trans-4a-Methyl-decahydronaphthalene	Polycyclic Alkane	***−6.6***	***−7.2***	***−6.6***
Undecane	Alkane	−5.6	−6.0	−5.8

Italicized Vina scores indicate that the ligands exhibit higher binding affinity to the receptor than nepetalactones and DEET.
